# Herbicide 2,4-dichlorophenoxyacetic acid interferes with MAP kinase signaling in *Fusarium graminearum* and is inhibitory to fungal growth and pathogenesis

**DOI:** 10.1007/s44154-023-00109-x

**Published:** 2023-08-15

**Authors:** Kaili Duan, Qifang Shen, Yu Wang, Ping Xiang, Yutong Shi, Chenfei Yang, Cong Jiang, Guanghui Wang, Jin-Rong Xu, Xue Zhang

**Affiliations:** 1 State Key Laboratory of Crop Stress Biology for Arid Areas, College of Plant Protection, Northwest A&F University, Yangling, 712100 Shaanxi China; 2 Department of Botany and Plant Pathology, Purdue University, West Lafayette, IN 47907 USA

**Keywords:** Fungal pathogenesis, MAP kinase pathways, 2,4-D, *Fusarium graminearum*

## Abstract

Plant hormones are important for regulating growth, development, and plant-pathogen interactions. Some of them are inhibitory to growth of fungal pathogens but the underlying mechanism is not clear. In this study, we found that hyphal growth of *Fusarium graminearum* was significantly reduced by high concentrations of IAA and its metabolically stable analogue 2,4-dichlorophenoxyacetic acid (2,4-D). Besides inhibitory effects on growth rate, treatments with 2,4-D also caused significant reduction in conidiation, conidium germination, and germ tube growth. Treatments with 2,4-D had no obvious effect on sexual reproduction but significantly reduced *TRI* gene expression, toxisome formation, and DON production. More importantly, treatments with 2,4-D were inhibitory to infection structure formation and pathogenesis at concentrations higher than 100 µM. The presence of 1000 µM 2,4-D almost completely inhibited plant infection and invasive growth. In *F. graminearum*, 2,4-D induced ROS accumulation and FgHog1 activation but reduced the phosphorylation level of Gpmk1 MAP kinase. Metabolomics analysis showed that the accumulation of a number of metabolites such as glycerol and arabitol was increased by 2,4-D treatment in the wild type but not in the *Fghog1* mutant. Transformants expressing the dominant active *FgPBS2*^S451D T455D^ allele were less sensitive to 2,4-D and had elevated levels of intracellular glycerol and arabitol induced by 2,4-D in PH-1. Taken together, our results showed that treatments with 2,4-D interfere with two important MAP kinase pathways and are inhibitory to hyphal growth, DON biosynthesis, and plant infection in *F. graminearum*.

**Supplementary Information:**

The online version contains supplementary material available at 10.1007/s44154-023-00109-x.

## Highlights

Herbicide 2,4-D was found to inhibit growth, DON biosynthesis, and plant infection in *Fusarium graminearum*. Whereas ROS accumulation and FgHog1 activation were stimulated, Gpmk1 phosphorylation was reduced by 2,4-D treatments.

**Supplementary Information:**

The online version contains supplementary material available at 10.1007/s44154-023-00109-x.

## Introduction

The filamentous ascomycete *Fusarium graminearum* is a major causal agent of Fusarium head blight (FHB) that affects the production of wheat, barley, and other grains and poses a threat to global food security (Bai and Shaner. 2004; Goswami and Kistler. 2004). This pathogen is a homothallic fungus that forms sexual fruiting bodies known as perithecia to survive on plant debris. It initiates infection of floral tissues of cereal crops with airborne ascospores that are ejected from perithecia. *F. graminearum* is also a producer of toxic secondary metabolites such as the trichothecene mycotoxin deoxynivalenol (DON) that often contaminates infested grains. As an inhibitor of eukaryotic protein synthesis, DON is an important virulence factor that is essential for spreading infectious growth through the rachis in infected wheat heads (Desjardins. 2003; Proctor et al. 1995a, b).

Genetic and molecular manipulations are relatively efficient in *F. graminearum*. To date, over a thousand of genes with various biochemical or biological functions, including those encoding protein kinases, transcription factors, and G-protein coupled receptors (GPCRs), have been characterized for their importance in hyphal growth, asexual and sexual reproduction, pathogenesis, secondary metabolism, and stress responses (Jiang et al. 2019; Son et al. 2011; Wang et al. 2011). Interestingly, all three mitogen-activated protein (MAP) kinase pathways play critical roles in regulating plant infection, DON biosynthesis, and sexual reproduction in *F. graminearum*, with each pathway having its unique functions (Jiang et al. 2018; Zhang et al. 2021). Whereas the *MGV1* MAP kinase (MAPK) is important for cell wall integrity and hyphal growth, the *GPMK1* pathway is required for penetration and invasive growth. Deletion of *MGV1* results in severe growth defects, which can be partially rescued by mutations in the *FgHOG1* high osmolarity glycerol (HOG) pathway that plays a key role in responses to hyperosmotic, oxidative, and other stresses (Ren et al. 2019). Like in other fungi, deletion of *FgHOG1* or other key components of the HOG pathway confers resistance to fludioxonil, a fungicide that overstimulates the FgHog1 MAPK and accumulation of compatible osmolytes such as glycerol, arabitol, and sorbitol (Van Thuat et al. 2012; Wen et al. 2022; Zheng et al. 2012). Although both *Fghog1* and *Gpmk1* deletion mutants are slightly reduced in growth rate, the *Gpmk1 Fghog1 mgv1* triple mutant grows faster than the *mgv1* mutant but is hypersensitive to various abiotic and biotic stresses (Ren et al. 2022).

Besides their functions in regulating growth and development, plant hormones such as auxin, salicylic acid (SA), jasmonic acid (JA), kinetin (KT), gibberellins (GAs), cytokinins, and brassinolides (BRs) play important roles in plant-pathogen interactions (Huang et al. 2020). In infection assays, exogenous application of SA, JA, GA, and Indole-3-acetic acid (IAA, the predominant natural auxin) increase resistance against FHB or spreading of infectious growth (Haidoulis and Nicholson. 2020; Petti et al. 2012). In contrast, treatments with KT disturb defense mechanisms and cell wall fortifications in the host and promote FHB severity (Buhrow et al. 2021). Although the underlying mechanisms are not clear, several plant hormones, including GA, IAA, and cytokinins, exhibit inhibitory effects on fungal growth in axenic cultures (Chanclud and Morel. 2016; Degani et al. 2015; Gupta et al. 2021). *F. graminearum* is known to produce IAA with the l-tryptophan (L-TRP)-dependent pathway (Luo et al. 2017). However, exogenous IAA is inhibitory to hyphal growth of *F. graminearum*, which is similar to reports in other plant pathogenic fungi (Degani et al. 2015; Luo et al. 2016; Nicastro et al. 2021; Svoboda et al. 2021). The herbicide 2,4-dichlorophenoxyacetic acid (2,4-D) shares structural similarity with IAA and acts as a mimic of auxin. Its inhibitory effects on fungal growth also have been reported in *Umbelopsis isabelline* (Nykiel-Szymańska et al. 2018).

To determine the effects of different phytohormones or phytohormone-mimicking compounds on *F. graminearum* and use it to characterize the underlying mechanisms, we assayed the effects of different plant hormones or their analogs, including KT, ABA, IAA, 2,4-D, and GA, on *F. graminearum* and found that 2,4-D had the highest inhibitory effects on fungal growth. Treatments with 2,4-D also reduced virulence, DON production, and Gpmk1 phosphorylation but induced ROS accumulation and FgHog1 activation. Metabolite profiling analysis showed that the stimulatory effect of 2,4-D on the accumulation of glycerol and arabitol was abolished by deletion of *FgHOG1*. Overall, our results showed that treatments with 2,4-D are inhibitory to hyphal growth, DON biosynthesis, and plant infection in *F. graminearum*, partly by interfering with the Gpmk1 and FgHog1 MAPK kinase pathways.

## Results

### Treatments with high concentrations of 2,4-D and IAA significantly reduce hyphal growth

**Fig. 1:**
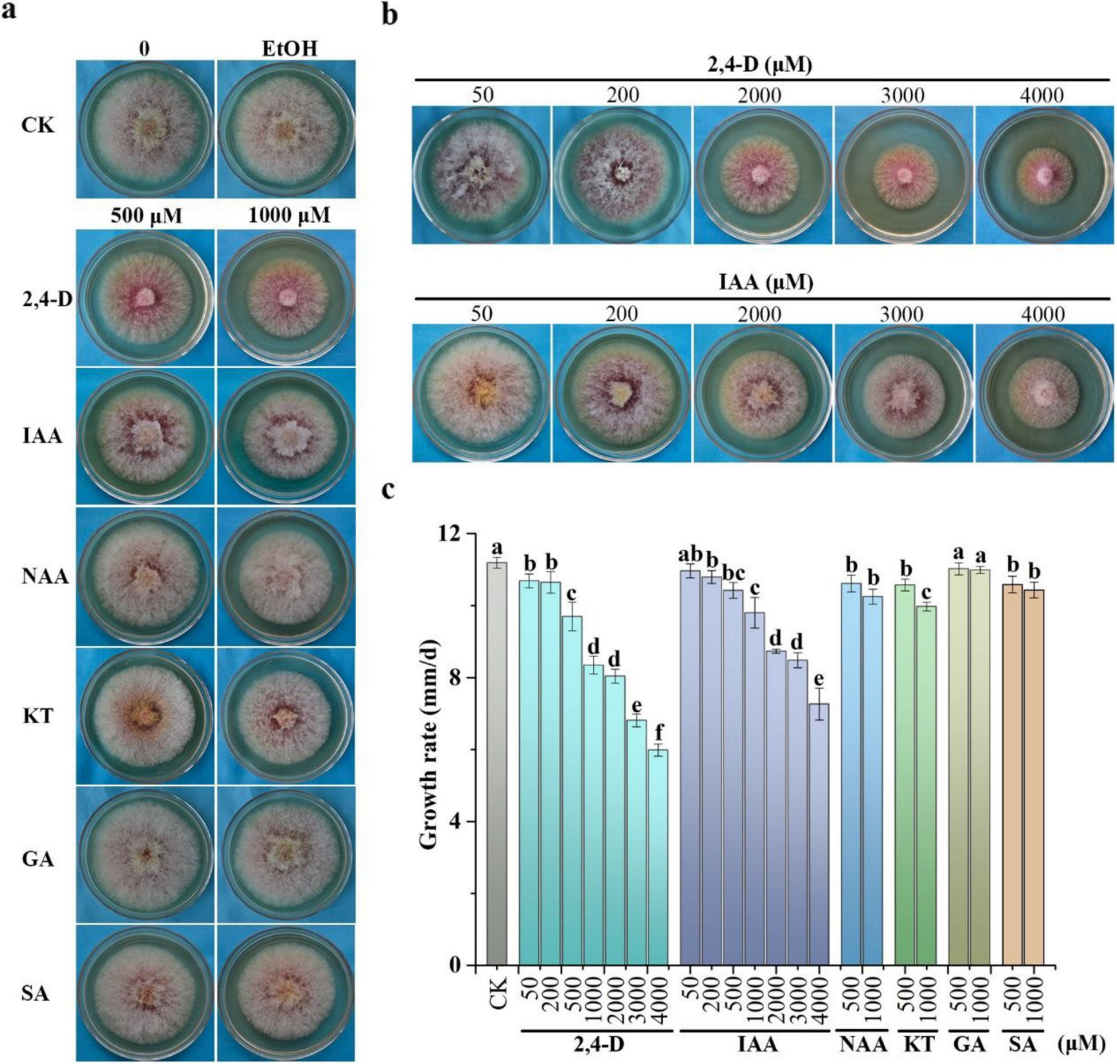
Assays for inhibitory effects of selected phytohormones and 2,4-D. **a** Three-day-old PDA cultures of PH-1 treated with 500 or 1000 μM of 2,4-D, IAA, NAA, KT, GA, or SA. The controls (CK) are regular PDA (0) and PDA with 0.4% ethanol (EtOH). **b** Three-day-old cultures of PH-1 with the addition of labelled concentrations of 2,4-D or IAA added to PDA. **c** The average growth rate of PH-1 on PDA plates with the labelled treatments was measured as the daily extension of colony radius. Mean and standard deviation were estimated with data from three independent replicates, with at least 3 culture plates in each replicate. Different letters indicate significant differences with the 0.4% ethanol control based on ANOVA analysis followed by Tukey’s range tests (*p* < 0.05)

To determine their effects on *F. graminearum*, we treated the wild-type strain PH-1 with several phytohormones or phytohormone-mimicking compounds, including 2,4-D, IAA, KT, NAA, gibberellin (GA) and SA. At the concentrations that affected growth in other fungi (Bönnighausen et al. 2019; Luo et al. 2016; Manzo-Valencia et al. 2016), treatments with GA had no significant or obvious effects, but the presence of 1000 µM KT caused a 10.8% reduction in growth rate in comparison with the untreated control. However, treatments with 1000 µM 2,4-D resulted in the most significantly reduction in growth rate of PH-1 (Fig. 1a). Even at 500 µM, 2,4-D was inhibitory to hyphal growth. Furthermore, colonies on cultures with 500 or 1000 µM 2,4-D were less fluffy (Fig. 1a), indicating inhibitory effects on aerial hyphal growth.

2,4-D is a metabolically stable analogue of IAA that regulates cell division, differentiation, and senescence in plants (Romero-Puertas et al. 2022). Because 1000 µM 2,4-D and IAA appeared to be more effective than 500 µM in inhibiting hyphal growth, we treated *F. graminearum* with higher concentrations of 2,4-D and IAA (Fig. 1b). In the presence of 2,000, 3,000, and 4,000 µM 2,4-D, growth rate was reduced 28.2%, 39.1%, and 46.5%, respectively, in comparison to the untreated control (Fig. 1c), indicating that 2,4-D has a dosage-dependent inhibitory effect on hyphal growth in *F. graminearum*. Similar dosage-dependent inhibition by IAA also was observed although it was less effective than 2,4-D at the same concentration (Fig. 1b). Growth rate was reduced 22.0%, 24.2% and 35.1% in cultures with 2,000, 3,000, and 4,000 µM IAA, respectively (Fig. 1c). Higher concentrations of 2,4-D also significantly reduced aerial hyphal growth, resulting the formation of colonies with limited aerial hyphae (Fig. 1b).

To further characterize its inhibitory effects, PH-1 was cultivated on PDA plates with lower concentrations of 2,4-D. In the presence of 50 and 200 μM 2,4-D, growth rate was slightly reduced (4.5% and 4.9%, respectively) but there was no obvious reduction in aerial hyphae (Fig. 1b, c). Growth on PDA with 50 and 200 μM IAA was similar to the no treatment control (Fig. 1b). We also measured the dry weights of hyphae grown in liquid YEPD cultures and showed that the presence of 500 and 1000 μM 2,4-D resulted in 18.3% and 28.6% reduction in fungal biomasses, respectively, in comparison with the untreated control (Table 1). These results confirmed the dosage-dependent inhibition of 2,4-D on vegetative growth in *F. graminearum*.

**Table 1:** Effects of 2,4-D on conidiation, hyphal growth, and plant infection

**2,4-D** (μM)	**Conidiation**^**A**^ (10^4^/ml)	**Dry weight**^**B**^ (mg)	**Disease index**^**C**^
CK	127.9 ± 8.7^a^	269.2 ± 4.0^a^	15.0 ± 2.0^a^
50	120.8 ± 10.9^a^	263.5 ± 5.5^a^	12.0 ± 1.3^ab^
200	113.1 ± 10.6^a^	240.4 ± 5.8^b^	11.0 ± 1.1^b^
500	72.0 ± 14.1^b^	219.9 ± 8.7^c^	0.4 ± 0.1^c^
1000	50.5 ± 8.5^c^	192.1 ± 10.2^d^	0.0 ± 0.0^c^

Mean and standard deviation were calculated with results at least three independent replicates. Different letters indicate significant differences based on ANOVA analysis followed by Tukey’s multiple range test (*p* < 0.05)

^**A**^Number of conidia per ml in 5-day-old CMC cultures of PH-1 with 0.4% ethanol (CK) or different concentrations of 2,4-D

^**B**^Dry weight was measured with vegetative hyphae harvested from 24 h YEPD cultures after lyophilization

^**C**^Disease index was rated as the number of diseased spikelets per head at 14 dpi. At least 10 wheat heads were examined in each treatment

### Treatments with 2,4-D affect conidiation and germination but not sexual reproduction

**Fig. 2:**
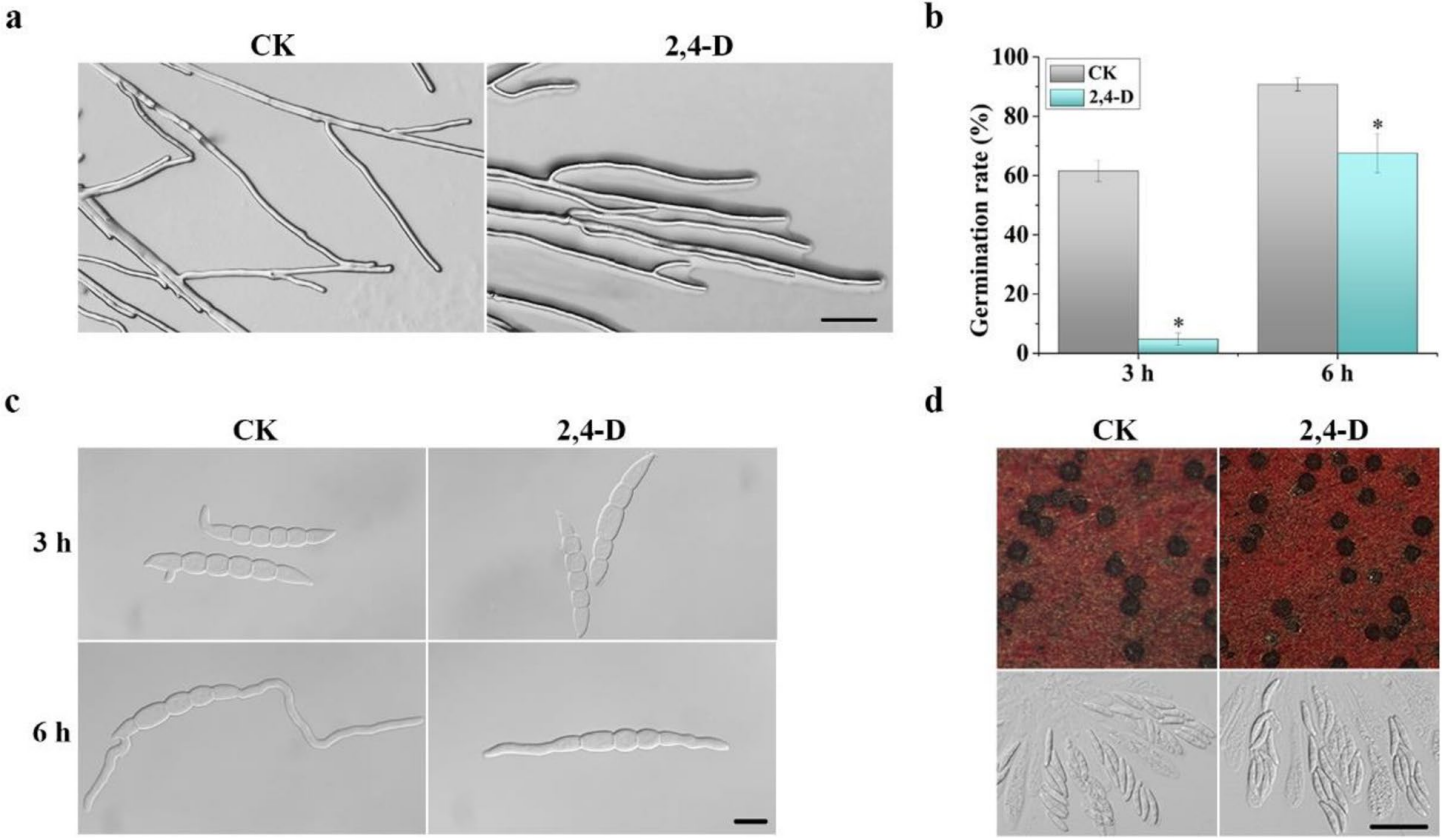
Effects of 2,4-D on hyphal tip growth, conidiation, and germination. **a** Hyphal tip growth and branching of PH-1 cultured on PDA plates with 2000 μM 2,4-D or 0.4% ethanol (CK). Bar = 50 μm. **b** Percentages of conidia germinated after incubation for 3 or 6 h in YEPD with 1000 μM 2,4-D or 0.4% ethanol (CK). Mean and standard deviation were estimated with data from three independent replicates. Asterisks indicate significant differences based on ANOVA analysis (*p* < 0.05). **c** Conidia of PH-1 incubated in YEPD with 1000 μM 2,4-D or 0.4% ethanol (CK) were examined for germination and germ tube growth after incubation for 3 and 6 h. Bar = 10 μm. **d** Mating cultures of PH-1 treated with or without 1000 μM 2,4-D were examined for perithecium formation and ascospore cirrhi at 8 days post-fertilization. Bar = 20 μm

When the edge of PDA cultures was examined, hyphae grew on the surface had smaller branching angles and were more aggregated in the presence of 2,4-D than the control (Fig. 2a), which may be directly related to reduced growth rate. In CMC cultures, conidiation (asexual reproduction) was significantly reduced by treatments with 500 or 1000 µM 2,4-D (Table 1). When assayed for conidium germination after incubation for 3 h in liquid YEPD medium, less than 5% conidia germinated in the presence of 1000 μM 2,4-D, which was significantly lower than 61.5% in the untreated control (Fig. 2b). Even after incubation for 6 h, 32.5% of conidia were not germinated and the ones germinated had shorter germ tubes in cultures with 1000 μM 2,4-D (Fig. 2c).

We then assayed the effect of 2,4-D on sexual reproduction. Abundant perithecia with asci and ascospores were formed on carrot agar plates with 1000 µM 2,4-D at 8 days post-fertilization (dpf) (Fig. 2d). No obvious differences were observed between mating plates with or without 2,4-D added to the carrot agar medium. These results indicated that treatments with 2,4-D have no obvious effect on sexual reproduction, which differs from its inhibition of vegetative growth and conidiation.

### Infectious growth is inhibited by high concentrations of 2,4-D

**Fig. 3:**
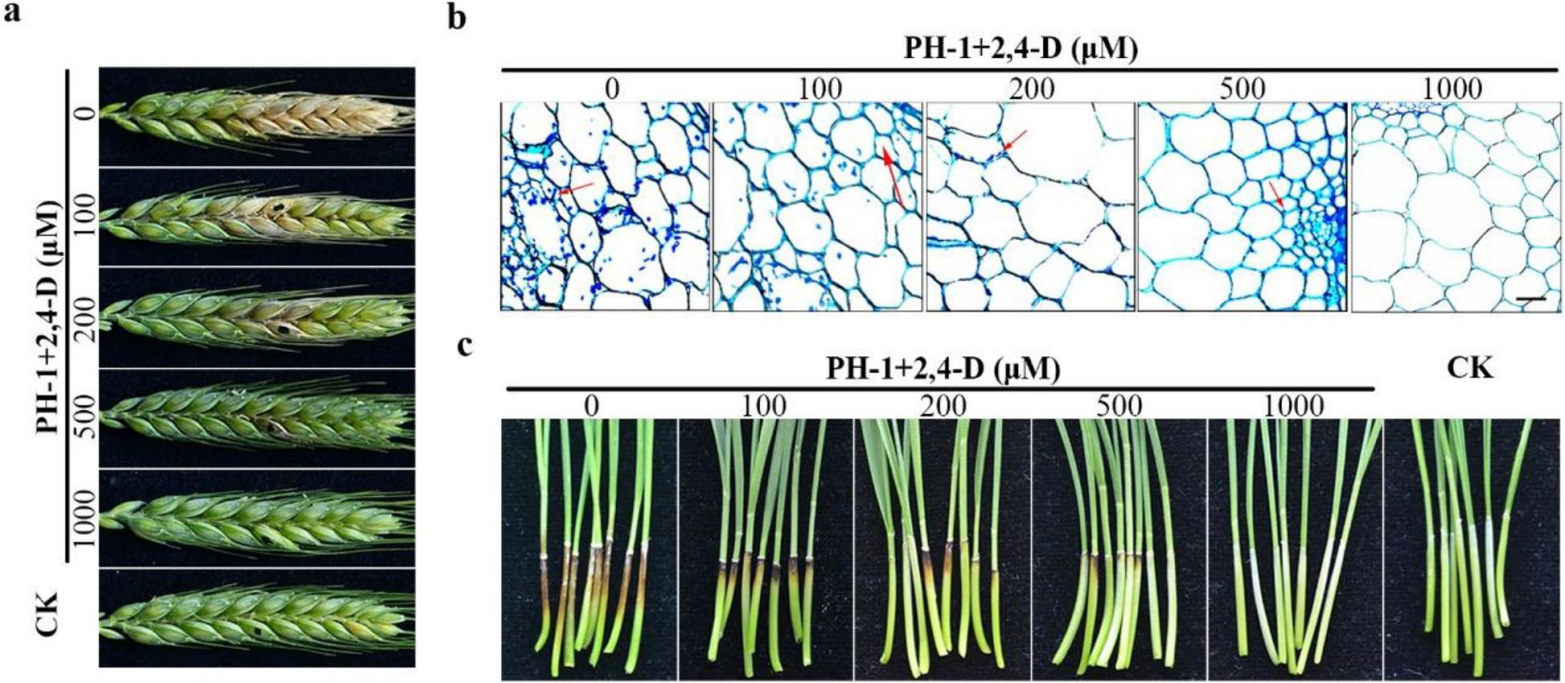
Inhibitory effects of 2,4-D on the virulence of *F. graminearum*. **a** Flowering wheat heads were drop-inoculated with conidium suspensions of PH-1 with the marked concentrations of 2,4-D and photographed at 14 dpi. Inoculations with 1000 μM 2,4-D (CK) were the control to show no detectable phytotoxic effects at this concentration. Black dots mark the inoculation site. **b** Thick sections of the rachis of wheat heads adjacent to the inoculated spikelets were examined for invasive hyphae (marked with arrows) at 5 dpi. Bar = 20 µm. Intercellular and intracellular growth of invasive hyphae were significantly reduced by 200 μM or higher concentrations of 2,4-D. **c** Wheat seedlings were wounded and inoculated with the marked conidium suspensions of PH-1 or 1000 μM 2,4-D (CK). Representative samples were photographed at 7 dpi

To determine its effect on fungal infection, flowering wheat heads were drop-inoculated with conidium suspensions of PH-1 containing different concentrations of 2,4-D. At 14 days post-inoculation (dpi), application of 1000 μM 2,4-D alone (no conidium control) did not cause necrosis and had no obvious effect on the development of inoculated wheat kernels (Fig. 3a), which is consistent with its herbicidal effects at concentrations higher than 2 g/L in the field (Wójcik et al. 2020). Nevertheless the virulence of PH-1 was reduced by 2,4-D in a concentration-dependent manner (Table 1). Whereas the presence of 1000 μM 2,4-D completely blocked *F. graminearum* infection, the disease index was reduced 26.7% and 97.3% by 200 and 500 μM 2,4-D, respectively.

When the rachis tissues next to the inoculated spikelets were examined, abundant infectious hyphae were observed in the control samples without 2,4-D. In samples treated with 200 or 500 μM 2,4-D, infectious hyphae were significantly reduced, particularly in the latter (Fig. 3b). Infectious hyphae were rarely observed in rachis tissues of samples treated with 1000 μM 2,4-D (Fig. 3b), which is consistent with the disease index being less than 0.5. These results indicate that 2,4-D is inhibitory to infectious growth and infection of wheat head tissues.

In infection assays with wheat coleoptiles, treatments with 200 and 500 μM 2,4-D also significantly reduced the virulence of PH-1 (Fig. 3c). The length of necrosis lesions was significantly reduced in the presence of 200 or 500 μM 2,4-D. Necrosis on wounded wheat leaf sheaths was rarely observed when inoculated with conidium suspensions with 1000 μM 2,4-D (Fig. 3c), which is consistent with results from wheat head infection assays. These results indicated that the application of high concentrations of 2,4-D inhibits the growth and spreading of infectious hyphae and reduces fungal virulence.

### Biosynthesis of DON is reduced by treatments with 2,4-D

**Fig. 4:**
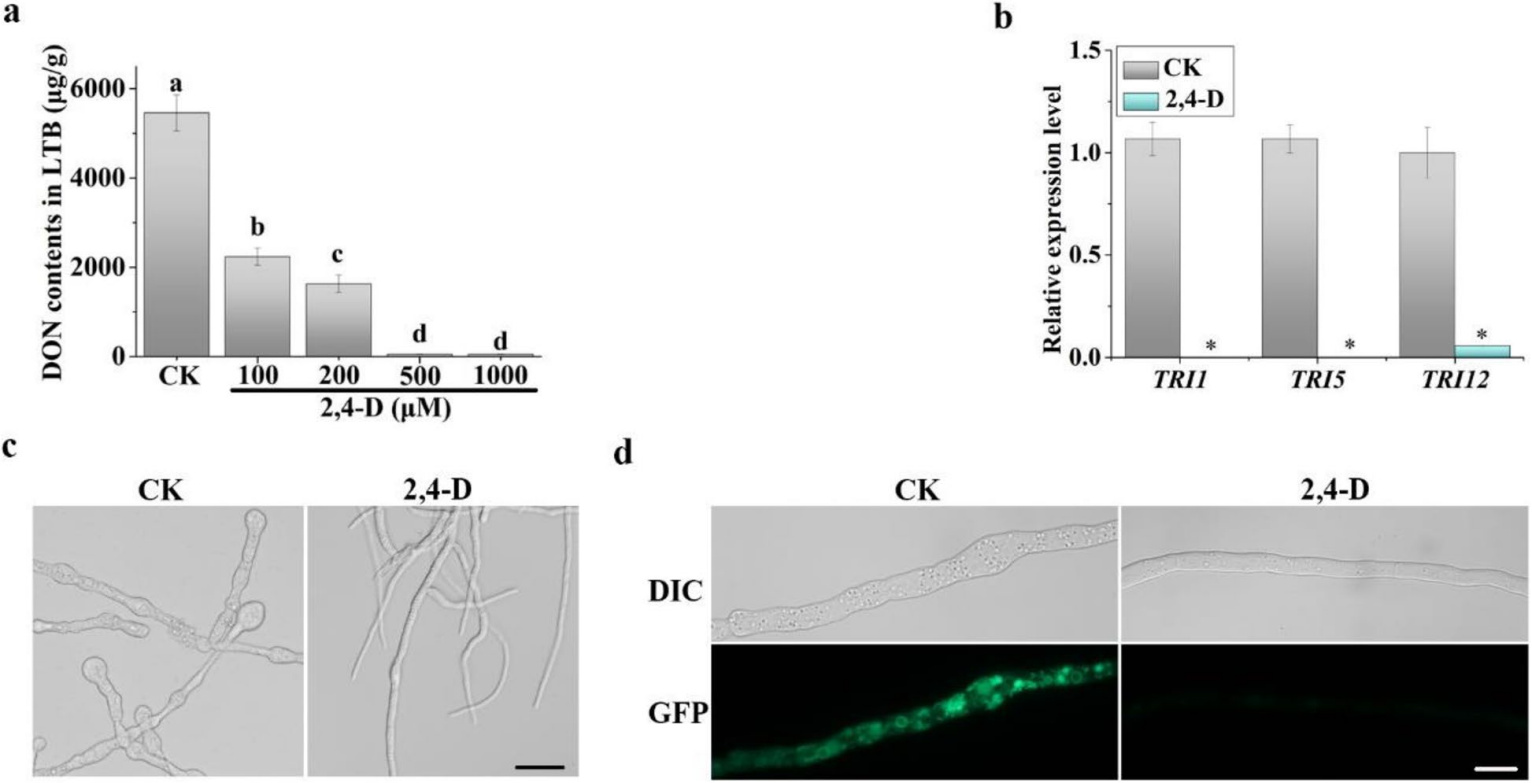
Effects of 2,4-D on DON production and *TRI* gene expression. **a** DON production in LTB cultures of PH-1 with 0.4% ethanol (CK), 100, 200, 500, or 1000 μM 2,4-D. Different letters indicate significant differences with CK based on ANOVA analysis followed by Tukey’s range tests (*p* < 0.05). **b** The expression of *TRI1*, *TRI5*, and *TRI12* was assayed by qRT-PCR with RNA isolated from 3-day-old LTB cultures with 0.4% ethanol (CK) or 500 μM 2,4-D. The relative expression level of each *TRI* gene in CK was set to 1. For both (**a**) and (**b**), mean and standard deviation were calculated with data from three independent replicates. Asterisks indicate significant differences with CK based on ANOVA analysis (*p* < 0.05). **c** Hyphae from three-day-old marked LTB cultures of PH-1 were examined for bulbous structures or swollen compartments. Bar = 20 μm. **d** Three-day-old LTB cultures of *TRI1*-*GFP* transformant treated were examined for the expression and localization of Tri1-GFP. Bar = 10 μm

Because DON is an important virulence factor in *F. graminearum* (Proctor et al. 1995a, b), we also assayed the effect of 2,4-D on DON biosynthesis in liquid trichothecene biosynthesis (LTB) medium (Gardiner et al. 2009). Treatments with 100 and 200 μM 2,4-D resulted in 59.1% and 70.2% reduction in DON production, respectively, in comparison with the ethanol solvent control (Fig. 4a). In the presence of 500 or 1000 μM 2,4-D, DON was barely detectable or only a low amount of DON was detected in 7-day-old LTB cultures (Fig. 4a).

To confirm the inhibitory effects of 2,4-D on DON biosynthesis, we selected the *TRI5*, *TRI1*, and *TRI12* genes for qRT-PCR assays. Whereas *TRI5* encodes the trichodiene synthase that catalyzes the first step of trichothecene biosynthesis, *TRI12* encodes an MFS transporter that functions as a trichothecene efflux pump (Alexander et al. 1999; Hohn and Beremand. 1989; Wang et al. 2018). The Tri1 calonectrin oxygenase is involved in late steps of trichothecene biosynthesis and it is a component of the toxisome (Menke et al. 2013). In qRT-PCR assays with RNA isolated from LTB cultures, the expression of the *TRI5* trichodiene synthetase gene was reduced over 1,428-fold by 500 μM 2,4-D in comparison with the ethanol solvent control (Fig. 4b). The expression level of the *TRI1* calonectrin oxygenase and *TRI12* transporter genes (Menke et al. 2012) also was significantly reduced by 2,4-D (Fig. 4b). Microscopical examination showed that the formation of intercalary swollen hyphal compartments in LTB cultures was significantly reduced by 500 μM 2,4-D (Fig. 4c). We also generated a transformant of PH-1 expressing the *TRI1*-GFP construct. Whereas GFP signals and toxisome formation were observed in untreated samples, treatments with 500 μM 2,4-D inhibited Tri1-GFP expression and toxisome formation (Fig. 4d). Taken together, 2,4-D is inhibitory to DON biosynthesis, likely by inhibiting *TRI* gene expression and toxisome formation.

### Treatments with 2,4-D cause oxidative stress

**Fig. 5:**
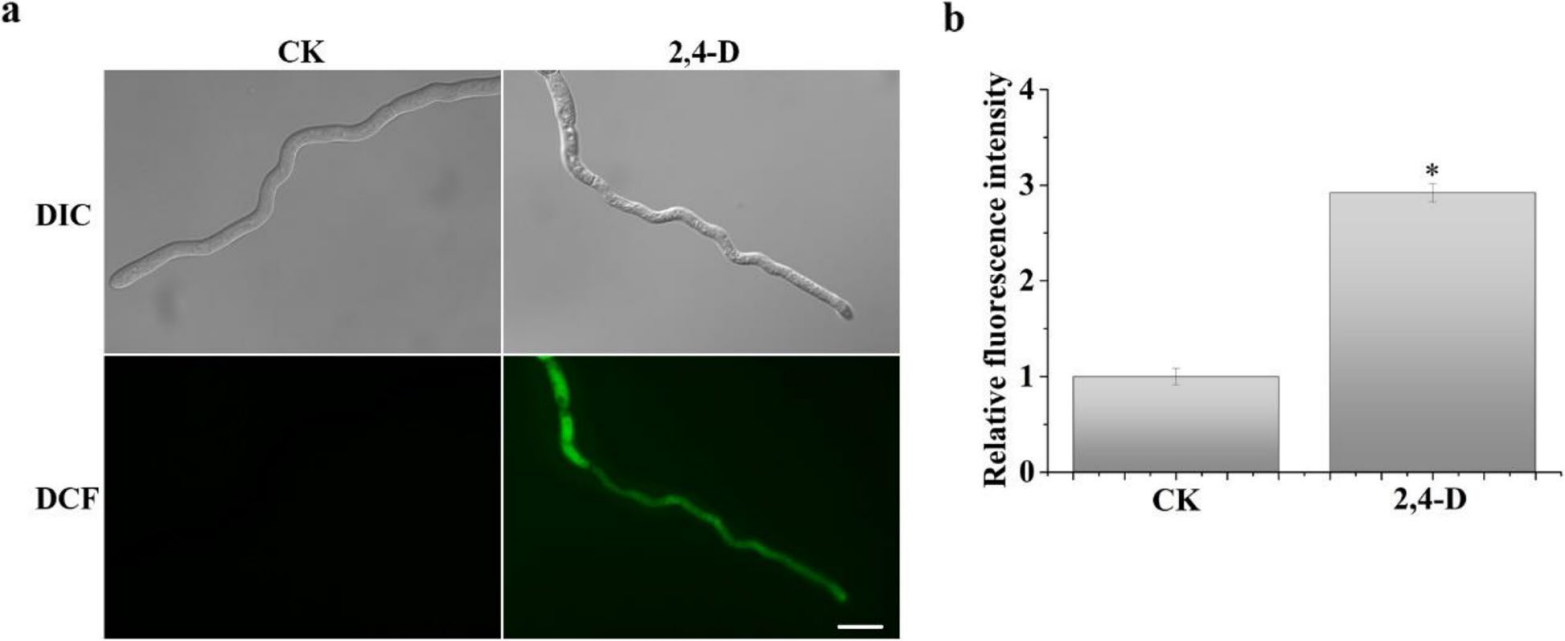
Accumulation of ROS in hyphae treated with 2, 4-D. **a** Germlings of PH-1 harvested from 8-h YEPD cultures were treated with 500 μM 2,4-D or 0.4% ethanol (CK) for 30 min and stained with dichloro-dihydro-fluorescein diacetate (DCFH-DA) before being examined by DIC and epi-fluorescence microscopy. Bar = 10 μm. Fluorescence signals indicate ROS accumulation in hyphae. **b** Relative fluorescence intensity of ROS accumulation in hyphae was measured with a micro-plate reader. The relative expression level of ROS in cultures with 0.4% ethanol (CK) was set to 1. Mean and standard deviation of the fluorescence intensity were estimated with data from three (*n* = 3) independent replicates. Asterisk indicate significant differences based on ANOVA analysis (*p* < 0.05)

Treatments with 2,4-D have been reported to induce membrane and oxidative stress in plants and the fungus *Umbelopsis isabellina* (Bernat et al. 2018a, b; Grossmann. 2010). To determine whether 2,4-D induced ROS in *F. graminearum*, we assayed ROS accumulation in conidia and germlings treated with 2,4-D by staining with dichlorodihydrofluorescein diacetate (DCFH-DA), a cell-permeable indicator for ROS (Liu et al. 2010). Intense green fluorescence signals were observed in the cytoplasm of germ tubes treated with 2,4-D but not in the control samples (Fig. 5a). In addition, we assayed the level of fluorescence signals based on excitation wavelength at 480 nm and emission wavelength at 530 nm (Oparka et al. 2016). The fluorescent signal in hyphae treated with 2,4-D was 2.9-fold higher than that of the untreated control (Fig. 5b). These observations showed that treatments with 2,4-D promoted the production of intracellular ROS in *F. graminearum.*

### The activation of FgHog1 MAP kinase is stimulated by 2,4-D

**Fig. 6:**
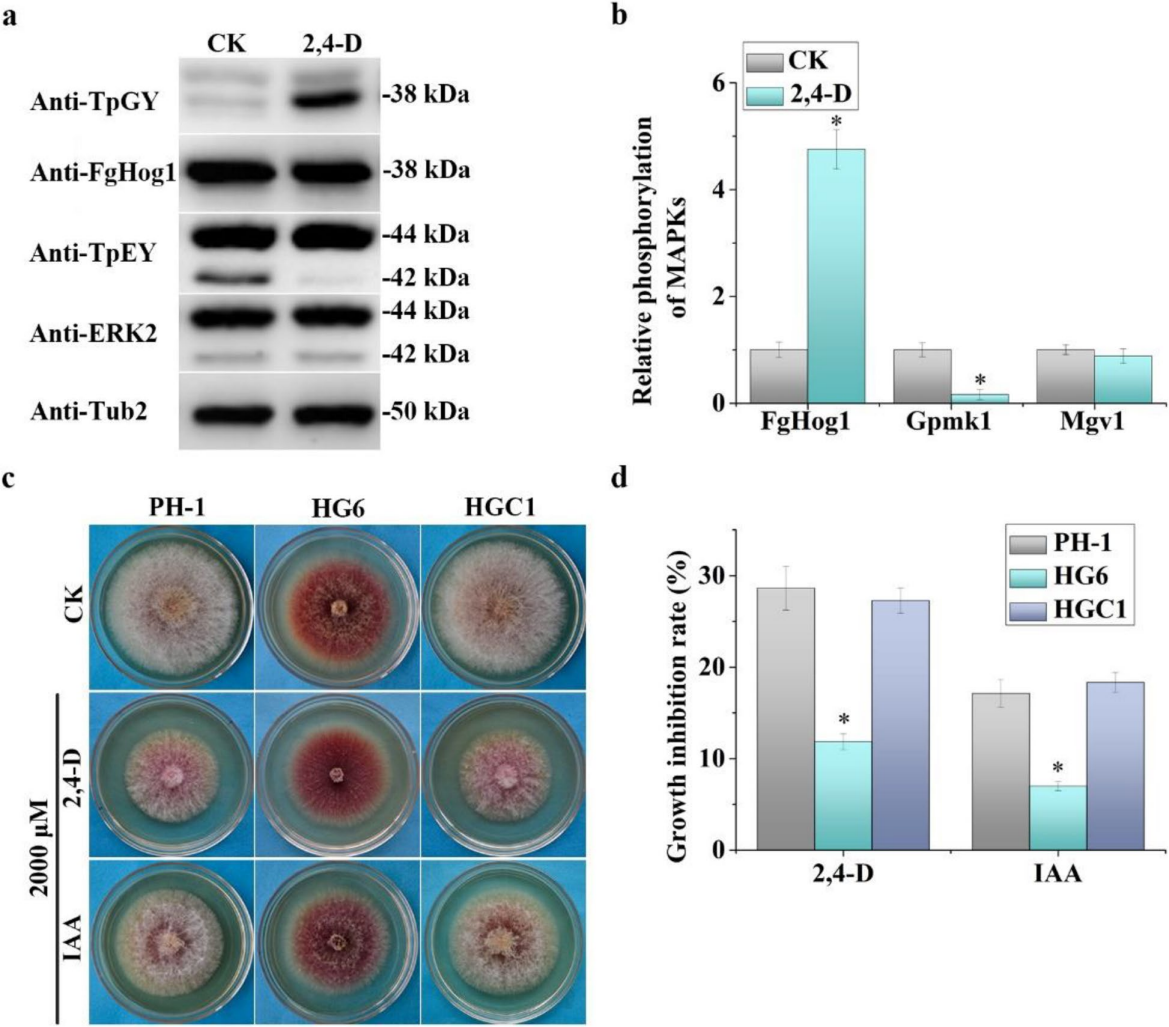
The effects of 2,4-D on MAP kinase activation and the *Fghog1* mutant. **a** Western blots of total proteins isolated from hyphae of PH-1 treated with 0.4% ethanol (CK) or 500 μM 2,4-D treatment for 30 min were detected with the anti-TpGY phosphorylation-specific, anti-FgHog1, anti-TpEY phosphorylation, anti-ERK2, or anti-Tub2 antibodies. Molecular weights of FgHog1, Mgv1, Gpmk1, and Tub2 are 38-, 44-, 42- and 50-kDa, respectively. **b** The densities of the FgHog1, Gpmk1, and Mgv1 bands were analyzed with Image J Software to estimate changes in their phosphorylation levels. For each MAP kinase, its relative phosphorylation level in hyphae treated with 0.4% ethanol (CK) was arbitrarily set to 1. **c** Three-day-old PDA cultures of PH-1, *Fghog1* mutant (HG6), and *Fghog1/FgHOG1* complemented (HGC1) strains with the addition of labeled concentrations of 2,4-D or IAA. **d** Colony radium of the same cultures was measured to estimate the growth inhibition rate by marked treatments as (1-mean colony radius of each treatment divided by that of the control) × 100%. For (**b**) and (**d**), mean and standard deviation were estimated with data from three independent replicates, with three culture plates in each replicate. Asterisks indicate significant differences in comparison with the control based on ANOVA analysis (*p* < 0.05)

Like in other fungi, the HOG pathway plays a critical role in regulating stress responses in *F. graminearum* (Zhang et al. 2021). Therefore, we assayed the effect of 2,4-D on the expression and activation of the FgHog1 MAP kinase. In proteins isolated from vegetative hyphae of PH-1 treated with 500 µM 2,4-D for 30 min, the expression of FgHog1 was not affected but its activation was significantly increased when detected with an anti-TpGY antibody (Fig. 6a). In repeated tests, the phosphorylation level of FgHog1 was increased over fourfold in samples treated with 2,4-D (Fig. 6b), suggesting a stimulatory effect on the activation of the FgHog1 MAPK pathway. In comparison with treatments with 50, 500, and 1,000 µM 2,4-D, the stimulation of FgHog1 phosphorylation appeared to be concentration dependent (Fig. S1).

Because over-stimulation of FgHog1 has a detrimental effect on hyphal growth in *F. graminearum* (Zhang et al. 2021), we assayed the inhibitory effect of 2,4-D on the *Fghog1* mutant HG6 that had a reduced growth rate compared to PH-1 (Zheng et al. 2012). In PDA cultures with 2000 μM 2,4-D, the growth inhibition rate on the *Fghog1* mutant was 11.8%, which was significantly less than 28.6% on PH-1 (Fig. 6c, d), suggesting increased tolerance against 2,4-D in the mutant. As the control, the *Fghog1*/*FgHOG1* transformant HGC1 had similar sensitivities to 2,4-D as PH-1 (Fig. 6c). The growth inhibition rate was 27.3% in the complemented transformant (Fig. 6d). These results indicated that overstimulation of FgHog1 may play a role in conferring sensitivities to 2,4-D in *F. graminearum.*

### Treatments with 2,4-D reduces the phosphorylation level of Gpmk1 MAP kinase

Because of the stimulatory effects of 2,4-D on FgHog1, we also assayed its effects on the expression and activation of Gpmk1 and Mgv1, two MAP kinases with the TEY dual phosphorylation site. When detected with an anti-Erk2 antibody, the expression of Gpmk1 and Mgv1 was not affected by 2,4-D treatment. However, when assayed with the anti-TpEY antibody, the phosphorylation of Gpmk1 was significantly reduced by 2,4-D although it had no obvious effects on Mgv1 activation (Fig. 6a). In comparison with the untreated control, treatments with 2,4-D resulted in over sixfold reduction in the phosphorylation of Gpmk1 (Fig. 6b).

**Fig. 7:**
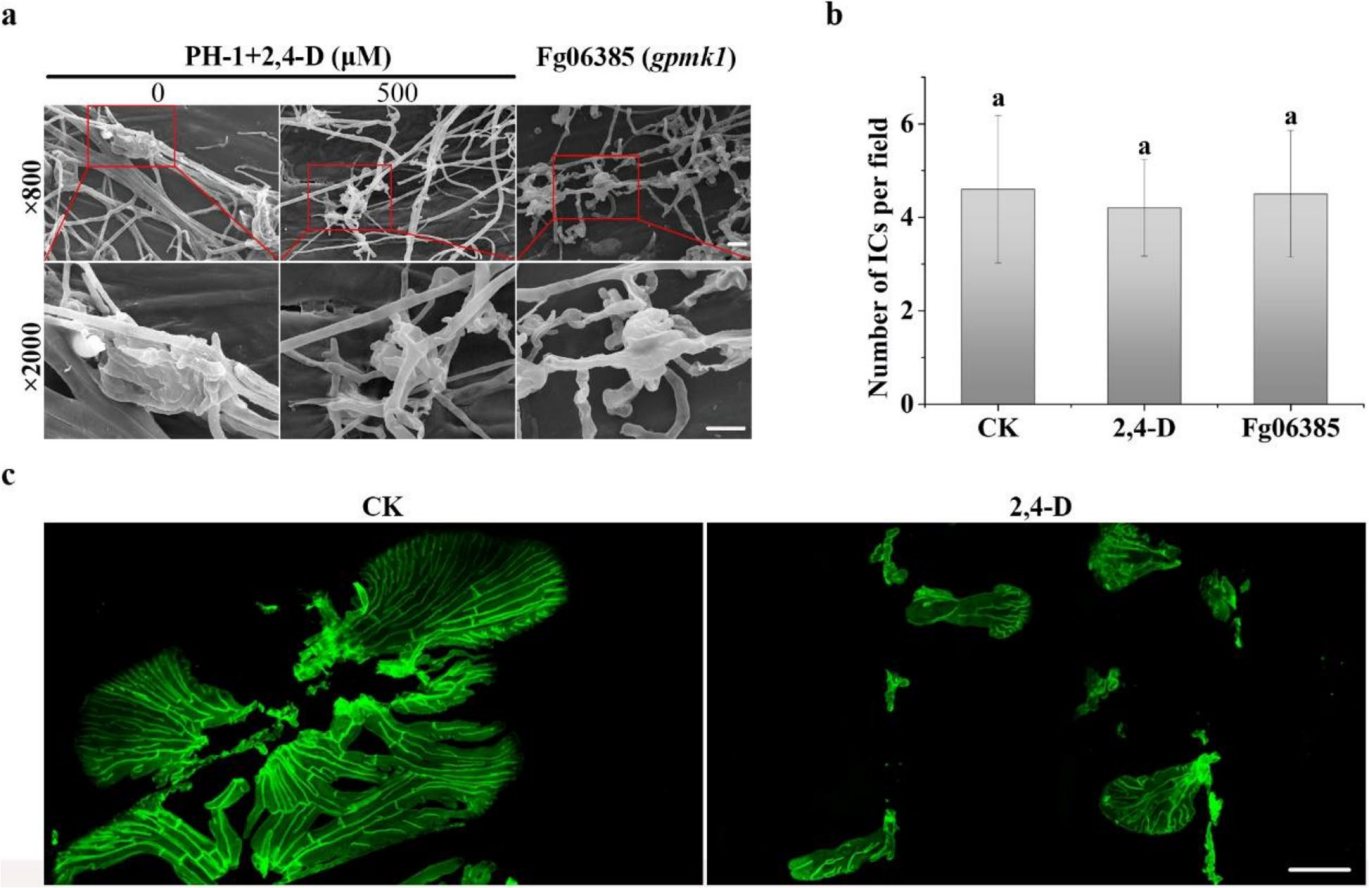
Effects of 2,4-D on initial plant infection processes. **a** Formation of infection cushions on lemmas by PH-1 treated with 0.4% ethanol (CK) or 500 μM 2,4-D and the *Gpmk1* mutant (Fg06385). The lower panels are the enlargement of the boxed areas in the upper panels. Bars = 10 µm. **b** The average number of infection cushions (ICs) in an examination field under × 800 magnification (SEM) in lemma samples inoculated with conidium suspension of PH-1 or *Gpmk1*. Mean and standard deviation were estimated with data from three independent replicates and subjected to ANOVA analysis followed by Tukey’s test (*p* < 0.05). **c** Wheat lemmas inoculated with PH-1 conidia treated with 0.4% ethanol (CK) or 500 μM 2,4-D were sampled at 72 hpi and examined by epifluorescence microscopy for coralloid invasive hyphae after staining with Alexa Fluor 488. Bar = 20 μm

In *F. graminearum*, the *Gpmk1* deletion mutant is non-pathogenic and its ortholog has a conserved function in regulating plant infection processes in different fungal pathogens (Jenczmionka et al. 2003; Xu. 2000). The inhibitory effects of 2,4-D on Gpmk1 activation may contribute to its inhibitory effect on plant infection. To test this hypothesis, we assayed the effect of 500 µM 2,4-D on the formation of infection structures on wheat lemmas. When examined by scanning electron microscopy (SEM), PH-1 treated with 2,4-D, similar to the *Gpmk1* deletion mutant, formed smaller infection cushions with less complex structures than the untreated control (Fig. 7a). However, treatments with 2,4-D appeared to have no obvious effects on the number of infection cushions formed by the wild type (Fig. 7b). Although infection cushions were not fully developed in the *gpmk1* mutant, deletion of *GPMK1* also lacked obvious effects on the number of infection cushions developed on lemmas (Fig. 7b). In addition, treatments with 2,4-D appeared to impair plant penetration and infectious growth in *F. graminearum.* In inoculated wheat lemmas, the growth of coralloid hyphae was significantly reduced by 2,4-D in comparison with the untreated control (Fig. 7c). Therefore, the inhibitory effect of 2,4-D on plant infection may be related to its inhibition on Gpmk1 activation in *F. graminearum*.

### The FgHog1 pathway regulates the accumulation of glycerol and sorbitol stimulated by 2,4-D

**Fig. 8:**
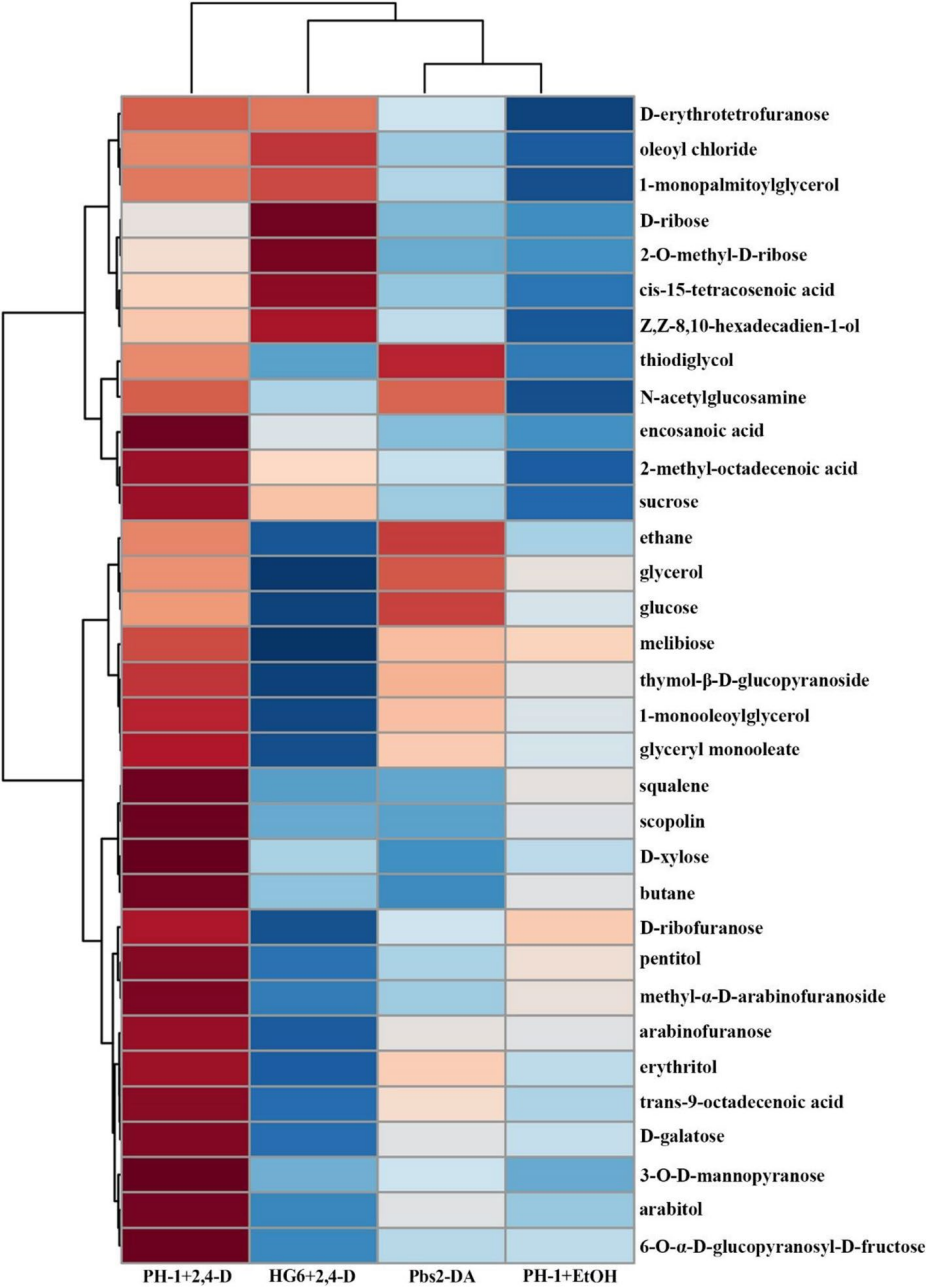
Effects of 2,4-D on metabolites in hyphae. Hierarchical clustering analysis (HCA) and heat map visualization of labelled metabolites that had at least twofold increase in hyphae of PH-1 treated with 500 μM 2,4-D in comparison with 0.4% ethanol (CK). Hyphae of the *Fghog1* mutant (HG6) treated with 500 μM 2,4-D and untreated hyphae of transformant expressing the *FgPBS2* dominant active allele (Pbs2-DA) were included for comparative analysis. The average content value of each metabolite in three biological replications (*n* = 3) was normalized and used for HCA analysis with Pearson correlation as the distance metric. Blue and red colors in heatmaps indicate the low and high abundance of metabolites, respectively

To determine the effect of 2,4-D on metabolism in *F. graminearum*, vegetative hyphae of PH-1 harvested from 20 h YEPD cultures were treated with 500 μM 2,4-D for 4 h. Metabolites were then extracted from treated hyphae after grinding in liquid nitrogen, desiccated, and derivatized with trimethylsilylimidazole (TMSI) and trimethylchlorosilane (TMCS) before GC–MS analysis as described (Smith and Bluhm. 2011; Zheng et al. 2012). Among the 96 metabolites identified (Table S1), 35 of them were significantly induced (over twofold, *p* < 0.05) by treatments with 500 μM 2,4-D in comparison with the control (Fig. 8), including glycerol and arabitol that are known to be accumulated in response to hyperosmotic stress under the control of the FgHog1 MAP kinase pathway in *F. graminearum* (Zheng et al. 2012).

**Fig. 9:**
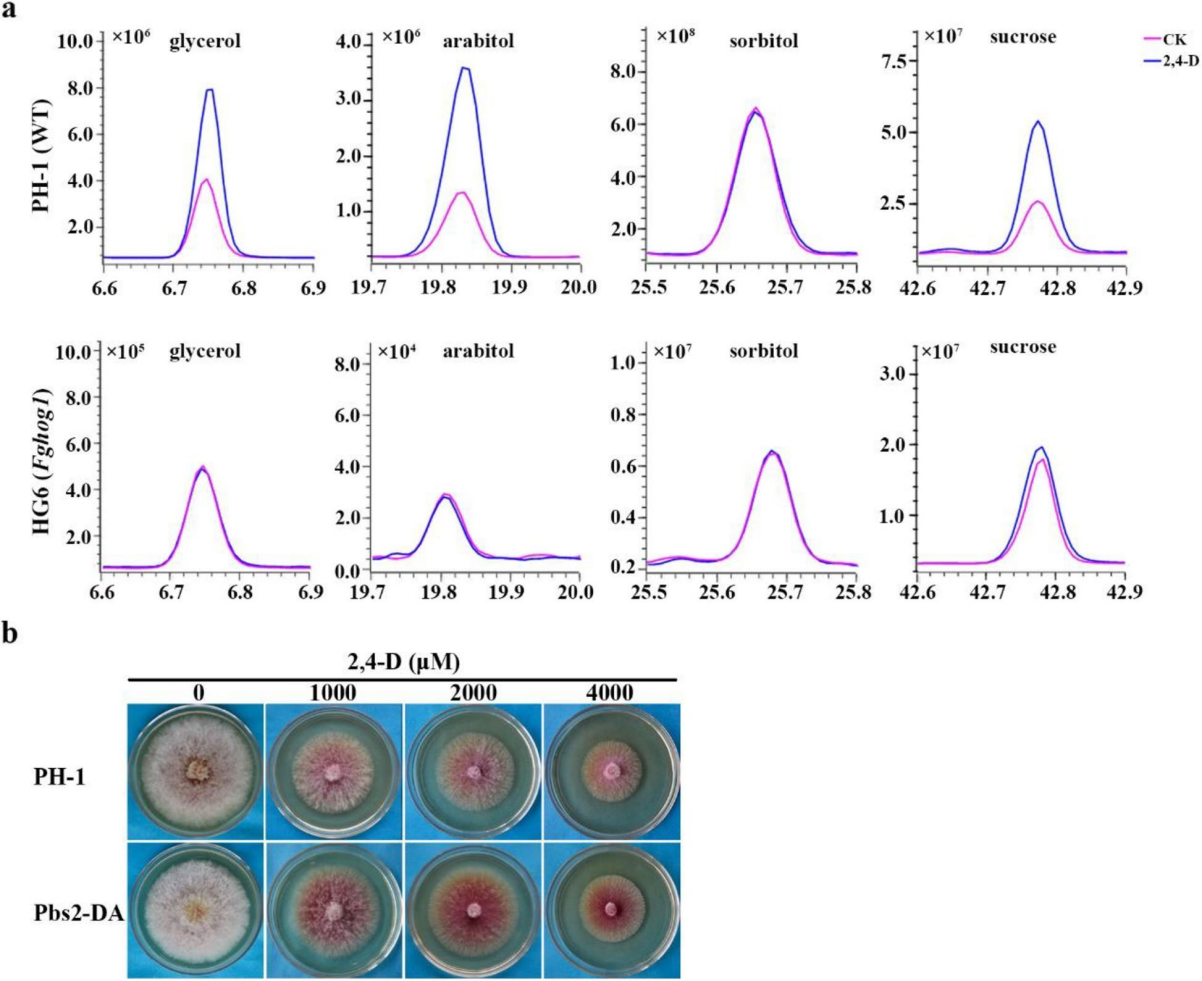
Assays for glycerol, arabitol, sorbitol, and sucrose in hyphae of PH-1 and the *Fghog1* mutant. **a** GC–MS fingerprint chromatograms of glycerol, sorbitol, inositol, and sucrose in extracts from hyphae of PH-1 and the *Fghog1* mutant (HG6) treated with 0.4% ethanol (CK) or 500 μM 2,4-D. The x-axis is the retention time (RT) in minutes. The height of each peak represents the total ion current. **b**. Three-day-old PDA cultures of PH-1 and the *FgPBS2*^S451D T455D^ transformant (Pbs2-DA) with the addition of labelled concentrations of 2,4-D

To determine whether 2,4-D stimulates the accumulation of glycerol and other compatible solvents via the FgHog1 pathway, we compared metabolites in the *Fghog1* mutant treated with 2,4-D. Among the 35 metabolites significantly stimulated by 2,4-D in PH-1, only 2 of them had increased over twofold levels in the *Fghog1* mutant after 2,4-D treatment (Fig. 8). Unlike in PH-1, the accumulation of glycerol and arabitol was not stimulated by 2,4-D in the *Fghog1* mutant although sucrose accumulation was not affected by *FgHOG1* deletion (Fig. 9a). Furthermore, we generated the *FgPBS2*^S451D T455D^ allele to mimic dominant active mutation and transformed it into PH-1. *FgPBS2* encodes the MAP kinase kinase that activates the FgHog1 MAP kinase. In comparison with PH-1, the *FgPBS2*^S451D T455D^ transformants were less sensitive to 2,4-D treatment (Fig. 9b). In PDA cultures with 1000 μM 2,4-D, the growth inhibition rate was 9.4% in the *FgPBS2*^S451D T455D^ transformant, which was significantly less than 23.6% in PH-1. Among the 35 metabolites significantly stimulated by 2,4-D in PH-1, 13 of them had increased over twofold levels in the *FgPBS2*^S451D T455D^ transformant in comparison with the untreated PH-1 control (Fig. 8). Taken together, these results suggested that stimulating the activation of the FgHog1 MAPK pathway likely contributes partially to changes in metabolites caused by 2,4-D in *F. graminearum*.

### Genes affected in hyphae by 2,4-D treatment

To determine the effect of 2,4-D treatment on gene expression, we isolated RNA from hyphae of PH-1 treated with 0.4% ethanol (solvent control) or 500 µM 2,4-D for 2 h and identified differentially expressed genes (DEGs, ≥ twofold changes) by RNA-seq analysis. In comparison with the control, 1,339 DEGs were up-regulated and 1,013 DEGs were down-regulated in PH-1 treated with 2,4-D (Table S2). KEGG enrichment analysis showed that DEGs up-regulated by 2,4-D were highly enriched for genes involved in carbohydrate, amino acid, and energy metabolism (Fig. S2). Consistent with its stimulation of ROS accumulation, the expression of *NOXA* and *NOXC* (Wang et al. 2014) was up-regulated more than twofold by 2,4-D. In contrast, the top two enriched categories in down-regulated DEGs are related to ‘cell growth and death’ and ‘protein folding, sorting and degradation’ (Fig. S2), which may be related to the oxidative stress and reduced growth rate caused by treatments with 2,4-D. Homologs of yeast *BUB2*, *CDC20*, *REC8*, *CDC6*, *CLB*, *RAD53* and *ORC1/2/5* that are involved in mitosis, septation, polarity establishment, and DNA replication are among the down-regulated DEGs by 2,4-D in the ‘cell growth and death’ category, which also include two Gα subunits known to regulate signal perception, propagation and growth in *F. graminearum* (Yu et al. 2008).

The intracellular glycerol level can be influenced by different metabolic pathways related to glycerol synthesis and metabolism (Foster et al. 2017). Although the expression of the glycerol-3-phosphate phosphatase gene (FGRRES_07096) was not affected, the haloacid dehalogenase (FGRRES_06381) and glycerol-phosphate dehydrogenase (FGRRES_03249) genes were up-regulated 1.9-fold and 14-fold, respectively, by 2,4-D treatment. In addition, the expression of triacyl/monoacyl-glycerol lipase genes FGRRES_02015, FGRRES_01603, and FGRRES_02944 was up-regulated over twofold by 2,4-D, suggesting a possible role of triglyceride breakdown in glycerol accumulation stimulated by this herbicide. For increased accumulation of arabitol, the expression of arabitol dehydrogenase genes was not affected but the ribulokinase gene (FGRRES_01430) was upregulated over twofold by 2,4-D treatment.

## Discussion

Plant hormones regulate a variety of physiological processes including growth, development, biotic and abiotic stress response. In this study, we found that treatments with KT, GA, and SA at concentrations reported to be inhibitory to growth in other fungi (Bönnighausen et al. 2019; Luo et al. 2016; Manzo-Valencia et al. 2016; Takeda et al. 2015) had no obvious effects on hyphal growth in *F. graminearum*. However, treatments with IAA and its analog 2,4-D were inhibitory to hyphal growth in a dose-dependent manner, with the latter being more metabolically stable and effective on *F. graminearum*. Significant inhibition on hyphal growth was observed when the concentration of 2,4-D was over 500 μM, but only when IAA was over 2000 μM. Even at 50 μM, 2,4-D still had a minor inhibitory effect on fungal growth. The growth rate of *F. graminearum* was not affected by the presence of 50 μM IAA, a concentration higher than its normal physiological concentration as a plant hormone (Kidd et al. 2011).

Although growth inhibition by IAA at the concentration of 500 and 1000 μM has been reported in other fungi such as *U. maydis* (Reineke et al. 2008), inhibitory effects of 2,4-D on fungal growth has only been reported in *U. isabelline* and growth rate was only slightly reduced after treatments for 120 h (Nykiel-Szymańska et al. 2018). In this study, we showed that treatments with 2,4-D not only inhibited hyphal growth but also reduced conidium germination, germ tube growth, and DON production. Whereas its inhibition on germ tube growth may be directly related to inhibitory effects of 2,4-D on polarized tip growth, conidium germination involves the establishment of polarized growth, which may be also disturbed by 2,4-D treatment. One of the down-regulated DEGs by 2,4-D is FGRRES_05216, an ortholog of yeast *PEA2* that is involved in polarity establishment. The expression of *FGRRES_05766*, an ortholog of yeast *CDC43*, also was reduced over twofold. For its inhibitory effect on DON biosynthesis, 2,4-D significantly reduced the expression of *TRI5* and other *TRI* genes as well as toxisome formation. Like in eukaryotes, Rho GTPases play important roles in morphogenesis and conidiation in *F. graminearum* (Zhang et al. 2013)*.* Two of its GTPases, *RHO1* and *RHO4*, were down-regulated over twofold by 2,4-D treatment.

Treatments with higher concentrations of 2,4-D were inhibitory to plant infection in *F. graminearum*, which has not been reported in other fungi. In samples treated with over 500 μM 2,4-D, infectious hyphae were significantly reduced in rachis tissues, indicating inhibitory effects of 2,4-D on infectious growth. However, most of the inoculated wheat kernels had no FHB symptoms in the presence of 1000 μM 2,4-D, indicating that the initial infection processes are also inhibited. Besides of its inhibitory effects on hyphal growth that may directly contribute to reduced virulence, 2,4-D was found to inhibit the activation of Gpmk1 MAP kinase. Like in other plant pathogenic fungi, this MAP kinase pathway regulates various infection processes and is essential for plant infection in *F. graminearum* (Jenczmionka et al. 2003). Significant reduction in Gpmk1 activation by 2,4-D may be another critical factor for its inhibitory effects on fungal pathogenesis. In fact, treatments with 2,4-D had similar effects on infection cushion formation and growth of coralloid hyphae in wheat lemma tissues as deletion of *GPMK1*. Although the underlying mechanism is not clear, a reduction in Gpmk1 activation by 2,4-D may be caused by overstimulation of FgHog1 because MAP kinase pathways are known to crosstalk (Zhang et al. 2021). Nevertheless, it is also possible that Gpmk1 activation is reduced by 2,4-D via its other targets*,* independent of FgHog1, in *F. graminearum*.

In *U. isabelline*, treatments with 2,4-D induce membrane and oxidative stress (Bernat et al. 2018a, b). In *F. graminearum*, treatments with 2,4-D induced ROS accumulation and increased the phosphorylation level of FgHog1 MAP kinase. Consistent with the overactivation of FgHog1, the intracellular level of glycerol and arabitol was increased in response to 2,4-D treatment in the wild type. In *F. graminearum* and other fungi, the FgHog1 MAP kinase is important for regulating response to oxidative and hyperosmotic stresses as well as glycerol synthesis (Zhang et al. 2021). In comparison with the wild type, the inhibitory effect of 2,4-D on hyphal growth was reduced in the *Fghog1* deletion mutant. Furthermore, the *FgPBS2*^S451D T455D^ transformant was less susceptible to 2,4-D and had increased accumulation of glycerol and arabitol that were stimulated by 2,4-D in PH-1. Thus, overactivation of the FgHog1 MAP kinase by 2,4-D may contribute partially to its inhibitory effects on *F. graminearum*. Phenylpyrrol fungicides such as fludioxonil overstimulate the activation of the HOG pathway and cause hyphal tip burst due to the accumulation of high concentrations of compatible solvents such as glycerol (Zhang et al. 2002). Although hyphal growth and branching were affected, hyphal tip burst was not observed by 2,4-D treatment in *F. graminearum*, which may be due to only relatively low concentrations of intracellular glycerol were accumulated in hyphae treated with 2,4-D in comparison with fludioxonil treatment (Zhang et al. 2002).

Overall, our results showed that treatments with 2,4-D were inhibitory to *F. graminearum* on growth, asexual reproduction, DON production, and pathogenesis, which may be directly related to its overactivation of FgHog1 and suppression of Gpmk1, two MAP kinases important for these infection and developmental processes (Jenczmionka et al. 2003; Zheng et al. 2012). However, besides its effects on these two MAP kinase pathways, 2,4-D likely has other targets in *F. graminearum,* as treatments with 2,4-D have been shown to increase or suppresse the expression of over 2,000 genes. Many of these genes are functionally unknown or their orthologs are not known to be related to FgHog1 or Gpmk1 MAP kinase pathways in fungi. For examples, *FgHyd4* and *MYT3* genes that are important for hyphal growth or conidiation in *F. graminearum* were reduced over twofold by 2,4-D treatment (Kim et al. 2014; Shin et al. 2022). Treatments with 4,000 µg/ml 2,4-D also resulted in more severe colonial and aerial hyphal growth than deletion of *FgHOG1* or *GPMK1*. Therefore, it will be important to generate and characterize mutants that become resistant or tolerant to 2,4-D to better understand the underlying mechanisms for the inhibitory effects of this herbicide on fungal growth and plant infection.

## Materials and methods

### Strains and cultural conditions

The wild-type strain PH-1 (Cuomo et al. 2007), *Fghog1* mutant HG6 (Zheng et al. 2012), and transformants generated in this study were routinely cultured on potato dextrose agar (PDA) at 25℃ (Yin et al. 2020). To determine their inhibitory effects on fungal growth, 2,4-D (Coolaber, China), IAA (Aladdin, China), NAA (Aladdin, China), KT (Aladdin, China), GA (Aladdin, China), and SA (Aladdin, China) were added to the final concentration of 500 and 1000 µM to PDA plates. IAA and 2,4-D were dissolved in 100% ethanol as the stock solutions and assayed with 0.4% ethanol (v/v) as the untreated control. Hyphae collected from YEPD cultures treated with 2,4-D for 24 h were lyophilized for 24 h in a Heto Powerdryer PL3000 (Thermo, USA) and measured for dry weights as described (Mao et al. 2020). Conidiation was assayed in 5-day-old CMC as described (Hou et al. 2002). For sexual reproduction, aerial hyphae of 7-day-old carrot agar cultures with or without 1000 µM 2,4-D were pressed down with 0.1% Tween 20 and further incubated at 25℃ under black light. Perithecium formation and production of cirrhi were examined at 8 days post-fertilization. Protoplast preparation, transformation, and selection of transformants resistant to hygromycin were performed as described (Hou et al. 2002; Zhou et al. 2010).

### Generation of the *TRI1-GFP* and *FgPBS2*^*S451D T455D*^ transformants

The *TRI1* gene was amplified with primer pairs TRI1-GFP-F/TRI1-GFP-R (Table S3) and fused to GFP by overlapping PCR. The resulting *TRI1*-GFP construct was transformed into protoplasts of PH-1 to obtain *TRI1-GFP* transformants. To introduce the S451D and T455D mutations in *FgPBS2* that were equivalent to the S514D and T518D mutations in yeast *PBS2* (Tatebayashi et al. 2020), we first used overlapping PCR to introduce the S451D and T455D mutations with primer pairs FgPBS2-1F/FgPBS2-1R and FgPBS2-2F/FgPBS2-2R (Table S3). The resulting PCR products were cloned into plasmid pKNTG (Lou et al. 2021) and transformed into PH-1. Transformants resistant to hygromycin were verified by PCR and sequencing analysis for carrying the transforming *FgPBS2*^S451D T455D^ allele.

### Plant infection assays

Wheat heads of cultivar Xiaoyan22 (Kang et al. 2008) were inoculated with 10 μl of conidia suspensions (2 × 10^5^ spores/ml in sterile distilled water) with 0, 100, 200, 500, or 1000 µM 2,4-D as described (Jiang et al. 2019). Inoculated spikelets were examined at 14 dpi for diseased kernels to estimate the disease index. For assaying infectious growth, infected rachis tissues were sampled at 5 dpi and embedded in Spurr resin after fixation and dehydration as described (Jenczmionka et al. 2003; Jiang et al. 2016). Thick Sects. (1 mm) were stained with 0.5% (w/v) toluidine blue and examined with an Olympus BX-53 microscope (Olympus, Japan). To assay infection cushion formation, inoculated lemmas were sampled at 3 dpi, fixed with 4% (v/v) glutaraldehyde, and coated with gold–palladium before examination by scanning electron microscopy JSM-6360LV (JEOL, Japan) as described (Jiang et al. 2019). To observe infectious growth in lemma tissues, inoculated lemmas were sampled at 3 dpi, stained with Alexa Fluor 488, and examined by laser scanning confocal microscope LSM880 (Zeiss, Germany) as described (Becker et al. 2016). For infection assays with wheat coleoptiles, Xiaoyan22 seedlings were cut off the top portion and inoculated with conidium suspensions with 2,4-D at the wound sites (Wu et al. 2005). Necrotic lesions on leaf sheaths were examined at 7 dpi. Inoculations with 1000 μM 2,4-D without conidia suspensions was the control to show no detectable phytotoxic effects.

### Assays for the intracellular ROS level

Intracellular ROS in hyphae was assayed with the reactive oxygen species assay kit purchased from Beyotime Institute of Biotechnology (Haimen, Jiangsu, China) following the instruction provided by the manufacturer (Jia et al. 2006). In brief, hyphae of PH-1 cultured in YEPD for 8 h were treated with 500 µM 2,4-D or 0.4% ethanol for 30 min. Treated hyphae were harvested by filtration and resuspended in 10 μM 2,7-Dichlorodihydrofluorescein diacetate (DCFH-DA). After incubation for 30 min at 37 °C, hyphae were examined by epifluorescence microscopy. The fluorescence intensity of intracellular ROS was measured with the microplate reader Victor Nivo (PerkinElmer, USA) with the excitation wavelength at 480 nm and emission wavelength at 530 nm.

### MAPK phosphorylation assays

Vegetative hyphae of PH-1 were harvested from 12 h YEPD cultures and treated with 500 μM 2,4-D or 0.4% ethanol (v/v) for 30 min. Total proteins were then isolated with protein lysis buffer containing protease inhibitor cocktail (Sigma-Aldrich, USA), phosphatase inhibitor cocktails 2 (Sigma-Aldrich, USA) and phosphatase inhibitor cocktails 3 (Sigma-Aldrich, USA) and separated on 10% PAGE gels as described (Zhang et al. 2018). Western blots were detected with the anti-TpGY phosphorylation-specific (for detection of phosphorylated FgHog1 with the TGY dual phosphorylation site), anti-FgHog1, anti-TpEY phosphorylation-specific (for detection of phosphorylated Gpmk1 and Mgv1 MAP kinases with the TEY dual phosphorylation site), and anti-ERK2 antibodies (Cell Signaling Technology, USA) as described (Zhang et al. 2018, 2017) for assaying the phosphorylation and expression levels of FgHog1, Gpmk1, and Mgv1 MAP kinases. Detection with an anti-β-tubulin (anti-tub2) antibody was used as the protein loading control as described (Wang et al. 2019).

### Assays for the effects of 2,4-D on DON biosynthesis and *TRI* gene expression

For assaying its effects on DON production, 2,4-D was added to the final concentrations of 100, 200, 500, and 1000 µM to 2 ml LTB (Liquid trichothecene biosynthesis) cultures with 1 × 10^4^ conidia/ml as described (Liu et al. 2019). After incubation at 25 °C for 7 d, DON was extracted from LTB cultures and analyzed with a GCMS-QP2010 (Shimadzu, Japan) as described (Liang et al. 2021). Hyphae from 3-day-old LTB cultures were examined for swollen hyphal compartments and toxisome formation (Menke et al. 2012). For assaying *TRI* gene expression, RNA was isolated from hyphae collected from 3-day-old LTB cultures with the TRIzol reagent (Invitrogen, USA). The FastKing RT Kit (TIANGEN, China) was used to assay the expression of *TRI* genes and actin gene of *F. graminearum* (as the internal control) with the CFX96 Real-Time System (Bio-RAD, USA) (Jiang et al. 2016). The mean and standard deviation of relative expression levels were calculated with data from three independent biological replicates.

### RNA-seq analysis

For RNA-seq analysis, vegetative hyphae harvested from 12-h YEPD cultures were resuspended in 500 µM 2,4-D or 0.4% ethanol and incubated for 2 h. RNA samples were then isolated with the TRIzol reagent. RNA-seq libraries were prepared with the NEBNext Ultra Directional RNA Library Prep Kit (NEB, USA) following the manufacturer’s instructions and sequenced with Illumina HiSeq 2500 with the paired-end 2 × 150 bp model at the Novogene Bioinformatics Institute (Beijing, China). The resulting RNA-seq reads were mapped onto the reference *F. graminearum* genome (Lu et al. 2022) by HISAT2 (Kim et al. 2015) and analyzed for the number of reads mapped to each gene with featureCounts (Liao et al. 2014). The edgeRun package with the exactTest function (Dimont et al. 2015) was used to analyze differentially expressed genes. Kyoto encyclopedia of genes and genomes (KEGG) pathway enrichment analyses of DEGs were conducted in R software using the package GO plot (Sun et al. 2021).

### Analysis for metabolic fingerprinting

Hyphae harvested from 20 h YEPD cultures were resuspended in YEPD medium with 500 µM 2,4-D or 0.4% ethanol (control) and further incubated for 4 h (Zheng et al. 2012). Treated hyphae were collected by filtration, washed 3 times with sterilized distilled water, and lyophilized for 24 h with Heto Powerdryer PL3000. After grinding in liquid nitrogen, 50 mg hyphal powders were resuspended in 1.6 ml methanol (w/v) and homogenized by sonification for 30 min. The supernatant (1 ml) was transferred to a new tube after centrifugation at 3500 rpm for 10 min and dried in a Savant ISS110 SpeedVac (Thermo, USA). After derivatization with 50 μl TMSI and TMCS (100: 1) (Sigma, USA) as described (Smith and Bluhm. 2011), each sample was mixed with 1 ml isooctane and 1 ml ultrapure water. The top organic phase was isolated after centrifugation and filtered with 0.22 μm nylon membrane (Shimadzu, Japan). For each sample, 1 μl was injected into a GCMS-QP2010 Ultra (Shimadzu, Japan) coupled with a Rxi-5 ms column (30 m × 0.25 mm × 0.25 μm). Helium was used as the carrier gas at a constant flow rate of 1 ml/min with the injector temperature of 260℃ and a split ratio of 30:1 (Zheng et al. 2012). The GC–MS detection conditions were as described (Smith and Bluhm. 2011). Shimadzu software GCMS Postrun Analysis was used for comparison of chromatograms, integration of peaks, and compound identification as described (Barreto et al. 2011). Heatmap visualization of log2-transformed compounds peak areas was performed using online software of MetaboAnalyst 5.0 (https://www.metaboanalyst.ca/) (Spicer et al. 2017).

### Supplementary Information

**Additional file 1: Table S1.** Metabolic profiles of PH-1, the *Fghog1* mutant HG6, and *FgPBS2*^*S451D T455D*^ transformant Pbs2-DA.

**Additional file 2: Table S2.** Expression profiles of PH-1 treated with 0.4 % ethanol or 500 µM 2,4-D treatment.

**Additional file 3: Table S3.** Primers used in this study.

**Additional file 4: Fig. S1.** The effects of different concentrations of 2,4-D on FgHog1 activation. Western blots of total proteins isolated from vegetative hyphae of PH-1 treated with marked concentrations of 2,4-D for 30 min were detected with the anti-TpGY phosphorylation-specific and anti-FgHog1 antibodies. **Fig. S2.** KEGG enrichment analysis of genes affected by 2,4-D. KEGG enrichment analysis of the differentially expressed genes (DEGs) up-regulated (A) and down-regulated (B) in cultures treated with 500 µM 2,4-D. The numbers in brackets are the number of DEGs in each category.

## Data Availability

The RNA-Seq data used in this study are available in National Center for Biotechnology Information (NCBI) Sequence Read Archive (SRA) and can be accessed with Bio-Project ID PRJNA956133.
