# Supplementary material for: Herbicide 2,4-dichlorophenoxyacetic acid interferes with MAP kinase signaling in Fusarium graminearum and is inhibitory to fungal growth and pathogenesis
Source: Stress Biol. 2023 Aug 15;3(1):31. doi: 10.1007/s44154-023-00109-x (PMC10442047; doi:10.1007/s44154-023-00109-x)
Supplement: Supplementary file 3 — Additional file 3: Table S3. Primers used in this study. [file 44154_2023_109_MOESM3_ESM.pdf]

Table S3. Primers used in this study

Primer name	Sequence (5’-3’)
TRI1QRT-F	TCATCAAGCCCCTGCAGGAAGAG
TRI1QRT-R	CACCATTGAAGCAACTTGAATG
TRI5QRT-F	GATACAGAGGACGCCAAGAAG
TRI5QRT-R	CGAACGTTTGCCAGTTGTG
TRI12QRT-F	GCCAGAGCGATAACCAAAGT
TRI12QRT-R	GTCGCCCAAATCTATCCGTAAG
FgPBS2-1F	AGGGAACAAAAGCTGGGTACCGCGTTCCATCCAAAACTATCACC
FgPBS2-1R	ACAGCCGATGTTGTCTCGGGCTATATCGGCGACAAGG
FgPBS2-2F	CCTTGTCGCCGATATAGCCCGAGACAACATCGGCTGT
FgPBS2-2R	GAACAGCTCCTCGCCCTTGCTCACGGCGTCATTTGGGCTTGAGATAG
TRI1-GFP-F	AGGGAACAAAAGCTGGGTACCGCTATACTCGGCAGTCCTTTGC
TRI1-GFP-R	GAACAGCTCCTCGCCCTTGCTCACGGCGTCATTTGGGCTTGAGATAG
