# Supplementary material for: Herbicide 2,4-dichlorophenoxyacetic acid interferes with MAP kinase signaling in Fusarium graminearum and is inhibitory to fungal growth and pathogenesis
Source: Stress Biol. 2023 Aug 15;3(1):31. doi: 10.1007/s44154-023-00109-x (PMC10442047; doi:10.1007/s44154-023-00109-x)
Supplement: Supplementary file 4 — Additional file 4: Fig. S1. The effects of different concentrations of 2,4-D on FgHog1 activation. Western blots of total proteins isolated from vegetative hyphae of PH-1 treated with marked concentrations of 2,4-D for 30 min were detected with the anti-TpGY phosphorylation-specific and anti-FgHog1 antibodies. Fig. S2. KEGG enrichment analysis of genes affected by 2,4-D. KEGG enrichment analysis of the differentially expressed genes (DEGs) up-regulated (A) and down-regulated (B) in cultures treated with 500 µM 2,4-D. The numbers in brackets are the number of DEGs in each category. [file 44154_2023_109_MOESM4_ESM.pdf]

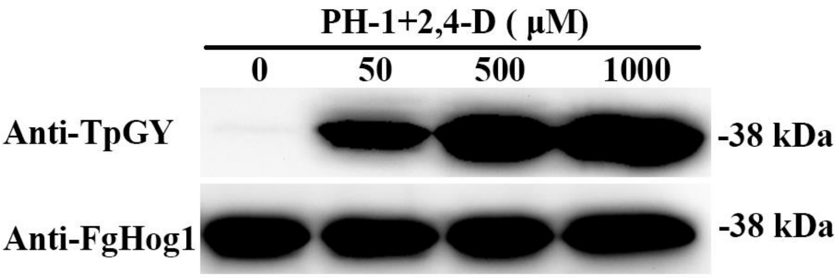

Fig. S1. The effects of different concentrations of 2,4-D on FgHog1 activation. Western blots of total proteins isolated from vegetative hyphae of PH-1 treated with marked concentrations of 2,4-D for 30 min were detected with the anti-TpGY phosphorylation-specific and anti-FgHog1 antibodies.

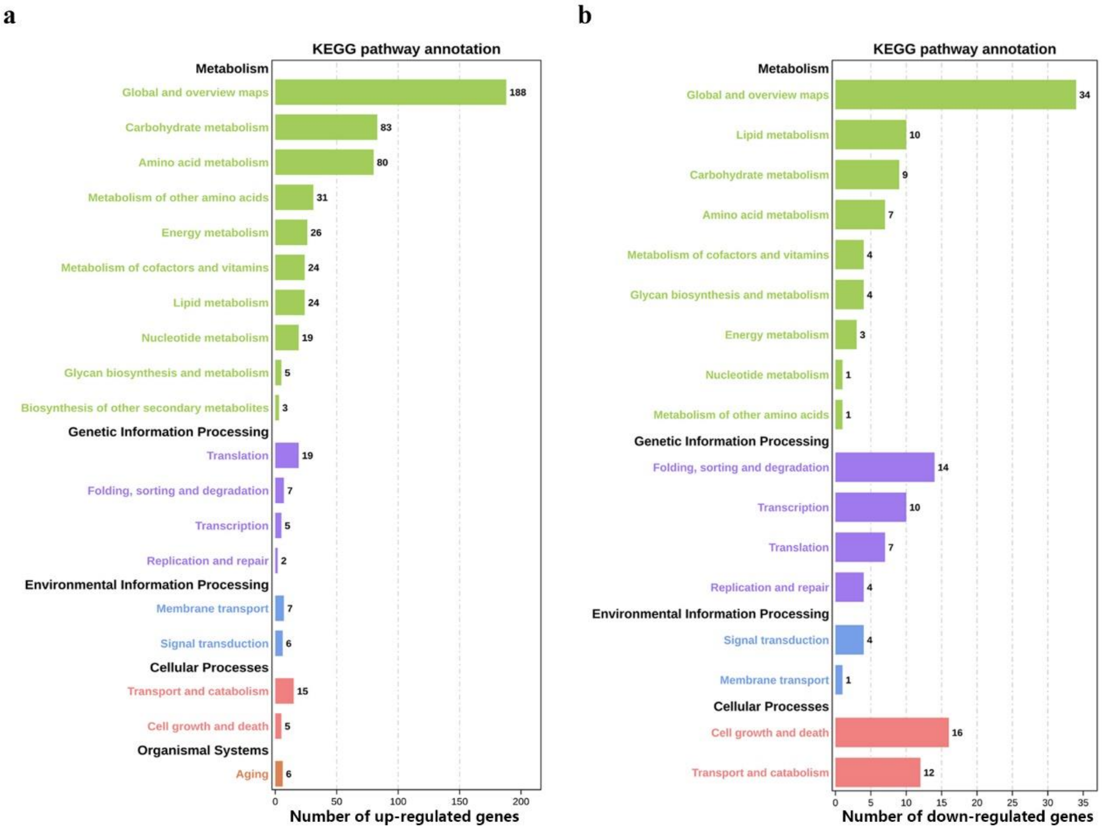

Fig. S2. KEGG enrichment analysis of genes affected by 2,4-D. KEGG enrichment analysis of the differentially expressed genes (DEGs) up-regulated (**a**) and down-regulated (**b**) in cultures treated with 500 μM 2,4-D. The numbers in brackets are the number of DEGs in each category.
